# Shedding Light on NF-κB Functions in Cellular Organelles

**DOI:** 10.3389/fcell.2022.841646

**Published:** 2022-05-10

**Authors:** Giovanna Carrà, Lidia Avalle, Laura Seclì, Mara Brancaccio, Alessandro Morotti

**Affiliations:** ^1^ Department of Clinical and Biological Sciences, University of Turin, Orbassano, Italy; ^2^ Department of Molecular Biotechnology and Health Sciences, Molecular Biotechnology Center, University of Turin, Turin, Italy

**Keywords:** NF-κB—nuclear factor kappa B, mitochondria, endoplasmic reticulum (ER), lysosome, Golgi, anticancer therapy

## Abstract

NF-κB is diffusely recognized as a transcriptional factor able to modulate the expression of various genes involved in a broad spectrum of cellular functions, including proliferation, survival and migration. NF-κB is, however, also acting outside the nucleus and beyond its ability to binds to DNA. NF-κB is indeed found to localize inside different cellular organelles, such as mitochondria, endoplasmic reticulum, Golgi and nucleoli, where it acts through different partners in mediating various biological functions. Here, we discuss the relationship linking NF-κB to the cellular organelles, and how this crosstalk between cellular organelles and NF-κB signalling may be evaluated for anticancer therapies.

## Introduction

The NF-κB family of transcription factors consists of five distinct proteins: RelA (p65), RelB, c-Rel, p100, and p105 (Zhang et al., 2017). This family is characterized by a rel homology domain (RHD) which allows its dimerization, binding to the DNA and the interaction with lκB inhibitors (Morotti et al., 2017). One of the most studied NF-κB dimers is the ubiquitously expressed p50 (arised from precursor proteins, p105)/RelA, which is involved in multiple cellular processes such as immune response, inflammation, proliferation, and apoptosis (Taniguchi and Karin, 2018). The activation of NF-κB is mediated by the canonical and non-canonical pathways. The canonical NF-κB pathway responds to several stimuli including growth factors, cytokines such as TNF-α and IL-1, inflammatory cell activation signals, viruses, microorganisms, and stress inducers. Activation of the canonical NF-κB pathway begins with the degradation of IκBα driven by its site-specific phosphorylation by a multi-subunit IκB kinase (IKK) complex composed of catalytic (IKKα and IKKβ) and regulatory (IKKγ; also known as NEMO) subunits. Proteasome-dependent degradation of IκBα allows NF-κB (p50/RelA or p50/c-Rel dimers) to translocate into the nucleus and regulate gene expression (Karin and Delhase, 2000; Hayden and Ghosh, 2008). In contrast, non-canonical NF-κB pathway responds only to specific stimuli such as ligands for a subgroup of TNF receptor (TNFR) family members, including LTβR, BAFFR, CD40 and RANK (Sun, 2012). NF-κB-inducing kinase (NIK) plays a key role in this pathway. NIK cooperates with IKKα in mediating p100 phosphorylation which in turn induces its processing generating a mature NF-κB2 (p52). Thus, NF-κB complex p52/RelB translocates in the nucleus (Xiao et al., 2001; Sun, 2011). Once in this compartment, NF-κB binds to κB target DNA elements and regulates gene transcription (Karin and Delhase, 2000; Hayden and Ghosh, 2008). A plethora of research has shown that NF-κB acts as a hub for different cellular signals, mediating responses in different cell compartments (Hayden and Ghosh, 2008; Hayden and Ghosh, 2012). In this review, we discuss the localization of NF-κB to various cell organelles and how it regulates organelle function by interacting with specific molecular partners. Moreover, we speculate on the relevance of these peculiar pathways and on their potential therapeutic implications.

## NF-κB and Mitochondria

### Role in Apoptosis

It is known that NF-κB can influence mitochondrial dynamics and respiratory chain and, reciprocally, mitochondria can pick up stress signals and convert them into cellular responses leading to NF-κB activation. Some studies have demonstrated the presence of the NF-κB RelA subunit and IκBα in the mitochondria (Albensi, 2019). The first evidence dates to 2001, when Bottero et al. found by immunoprecipitation that IκBα complexes with the mitochondrial adenine nucleotide translocator (ANT) (Bottero et al., 2001). This interaction led to the identification of a larger complex including also RelA, which is involved in the regulation of the apoptotic cascade. Intriguingly, the release of IκBα/RelA mitochondrial complex can inhibit the NF-κB-dependent upregulation of anti-apoptotic genes. IκBα can also be cleaved by Caspase 3, becoming a more stable NF-κB inhibitor (Levkau et al., 1999; Reuther and Baldwin, 1999). Pazarentzos et al. showed that IκBα regulates the mitochondrial outer membrane permeabilization (MOMP) and inhibits apoptosis by interacting with VDAC1–HKII complex. Indeed, IκBα knock-down or its reduction in the mitochondria can sensitize cells to apoptosis (Pazarentzos et al., 2014). In line with the above observation, we have recently identified lung tumors specimens in which NFKBIA (the gene coding for IκBα) is amplified. This amplification correlates not only with impaired mitochondrial metabolism, but also with lower chemosensitivity. Pharmacological or genetic approaches targeting IκBα induce oxidative metabolism and promote an increase in ROS level beyond the tolerated threshold, causing stress-dependent cell death (Carra et al., 2021). It has been demonstrated that hypoxia-stimuli induce a transient and rapid accumulation of RelA/IκBα complex into mitochondria in a STAT3-dependent manner. Mitochondrial STAT3 import is well documented and requires chaperones functions and STAT3 post-translational modifications, as phosphorylation and acetylation (Avalle and Poli, 2018). Similarly, RelA/IκBα complex requires the cooperation of several chaperones to enter mitochondria. Some data suggests a possible cooperation between STAT3 and RelA/IκBα in this process. Indeed, inhibition of STAT3 phosphorylation reduces its mitochondrial localization and the import of the RelA/IκBα complex. STAT3 is also implicated in the prevention of apoptosis in the mitochondria. Of note, a cooperation between RelA/IκBα complex and STAT3 has been described in the early stage of hypoxia (Ivanova and Perkins, 2019). Overall, mitochondrial NF-κB may mediate pro-oncogenic activities, inducing cell survival under stress stimuli, such as hypoxia.

### Role in Mitochondrial Gene Transcription

**FIGURE 1:**
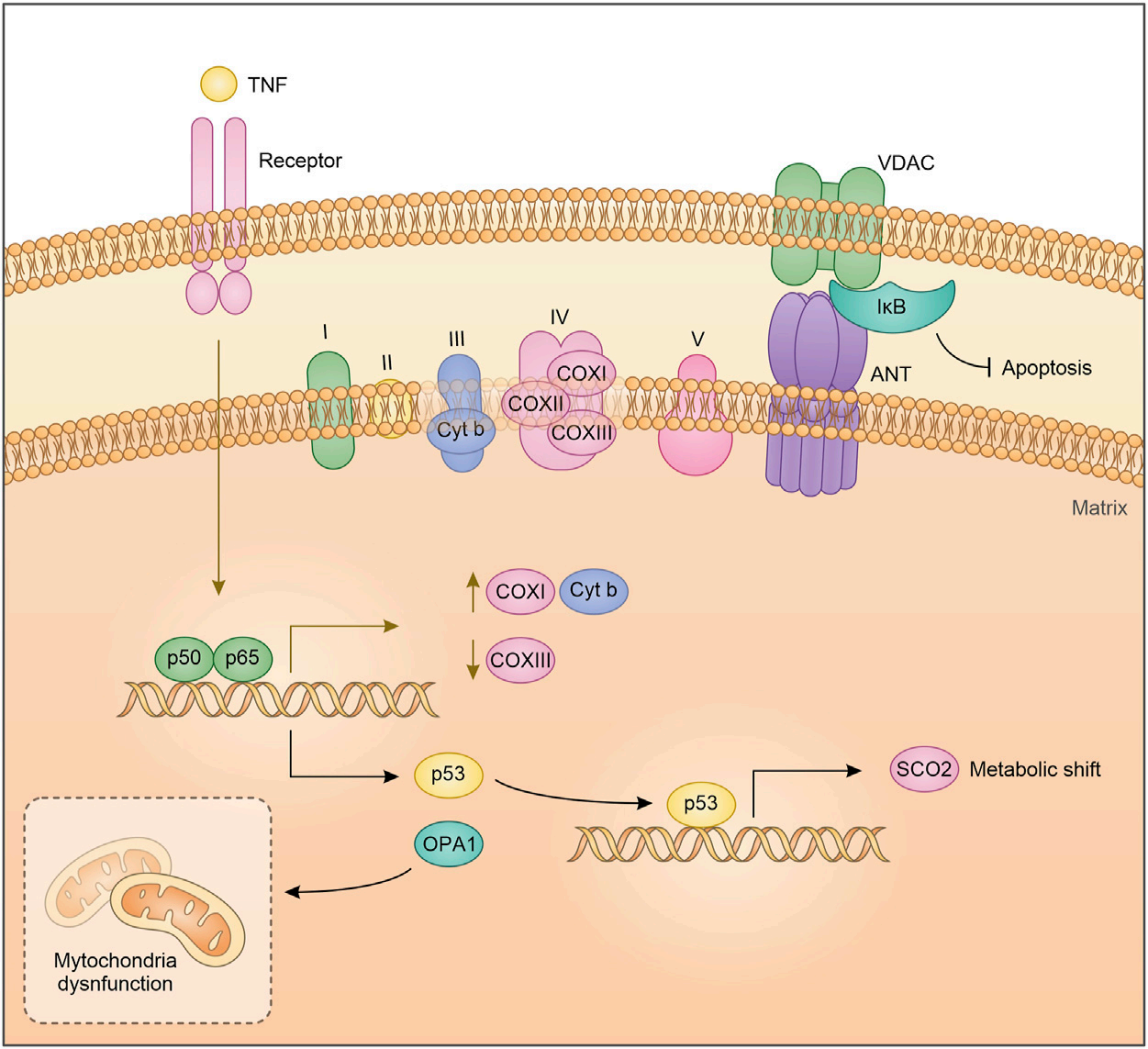
Schematic representation of NF-κB crosstalk in the mitochondria.

Despite the mitochondrial DNA retained different ancient prokaryotic properties, it is not inconceivable that the long endosymbiosis with the eukaryotic cell has made it sensitive to eukaryotic transcription factor regulation. Cogswell and colleagues proposed that NF-κB components in the mitochondria can bind mitochondrial DNA. EMSA analysis showed that NF-κB binds mitochondrial DNA mainly after TNFα treatment, suggesting that the pathway leading to NF-κB activation, as a nuclear transcription factor, can also regulate mitochondria DNA transcription (Cogswell et al., 2003). Particularly, Cogswell et al. showed an NF-κB-dependent decrease of mitochondrial Cox III expression in HT1080 cells after TNFα treatment. In agreement with this observation, it has been suggested that in prostatic carcinoma cells, treatment with TRAIL (Tumor necrosis factor–related apoptosis-inducing ligand) promotes NF-κB binding to mitochondrial DNA and decreases mitochondrial Cox III expression. Importantly, TRAIL does not induce a translocation of NF-κB from the cytoplasm to the mitochondria but appears to activate the pre-existing mitochondrial pool of NF-κB (Guseva et al., 2004). Consistent with the role as a regulator of mitochondrial genes, more recently it has been observed that NF-κB induces the transcriptional expression of COX I, and Cytb by binding to the D-loop region of mitochondrial DNA (Figure 1) (Psarra and Sekeris, 2008; Johnson and Witzel Perkins, 2011; Barshad et al., 2018). The discovery that transcription factors classically involved in nuclear gene regulation play a role in mitochondrial gene expression, highlights the existence of coordinated crosstalk between the nuclear and mitochondria gene expression. Of note, this interconnection could depend on extracellular mediators and cytokines.

### Role in Mitochondrial Dynamics and Respiration

By acting as a nuclear transcription factor, NF-κB mediates the expression of several proteins, some of which directly impact on mitochondrial dynamics. This is the case of OPA1, that is required for mitochondrial fusion and biogenesis. Indeed, OPA1 is drastically reduced in IKKα- and IKKα/β-deficient MEFs in contrast to IKKβ-deficient MEFs. Furthermore, IKKα- and IKKα/β-deficient MEFs showed significant fragmentation of mitochondria, a phenotype that is reverted by OPA1 expression, following the reintroduction of IKKα but not after NEMO expression (Laforge et al., 2016). Cancer cells increase their aerobic metabolism and reduce mitochondrial activity to aid their sustenance, a mechanism known as Warburg effect (Vander Heiden et al., 2009). In this respect, we have shown that IκBα down-modulation and therefore NF-κB activation in lung cancer cells, induces a mitochondrial unbalancing toward β-oxidation and an up-regulation of several key lipid metabolism genes, and in turn increases mitochondrial respiration (Carra et al., 2021). Accordingly, Mauro et al. identified NF-κB/RelA as a physiological regulator of mitochondrial respiration. Knock down of RelA enhanced ATP levels and glucose consumption while decreased oxygen utilization. Similar results were obtained in p53 silenced cells, recapitulating most of the metabolic changes obtained with the silencing of RelA, and indicating involvement of a p53-dependent mechanism. Reinforcing the interdependence between p53 and RelA in regulating mitochondrial metabolism, is the fact that inactivation of RelA upregulates p53-repressed basal genes, including GLUT1, GLUT3 and GLUT4 while downregulating SCO2, a p53 target gene. Lastly, ectopic expression of SCO2 or p53 reverted RelA cellular phenotype. Translating these observations on cancer, RelA-mediated metabolic rewiring can induce morphological characteristics typical of cancer cells, such as high proliferative rate and anchorage-independent growth (Figure 1) (Mauro et al., 2011).

### Mechanisms of Mitochondrial Translocation

The list of transcription factors that translocate and function in the mitochondria is exponentially increasing. Several results suggest that their function in this compartment is equally important to cell fate as their canonical activities in the nucleus (Szczepanek et al., 2012; Sepuri et al., 2017). How does NF-κB move into the mitochondrion? Very little is known on the mechanisms that mediate its translocation. Hypoxia is one of the stimuli proposed to trigger NF-κB mitochondrial entry and TOM40 and the matrix chaperone protein mortalin have been involved in its translocation. Moreover, inhibition of STAT3 blocks RelA and IκBα mitochondrial import (Johnson and Witzel Perkins, 2011; Ivanova and Perkins, 2019). However, considering the key role of NF-κB in the regulation of mitochondrial dynamics, it is plausible to hypothesize that a pool of NF-κB is always resident in the mitochondria and that specific signals may induce its activity.

## NF-κB and Nucleoli

### Role in Apoptosis

**FIGURE 2:**
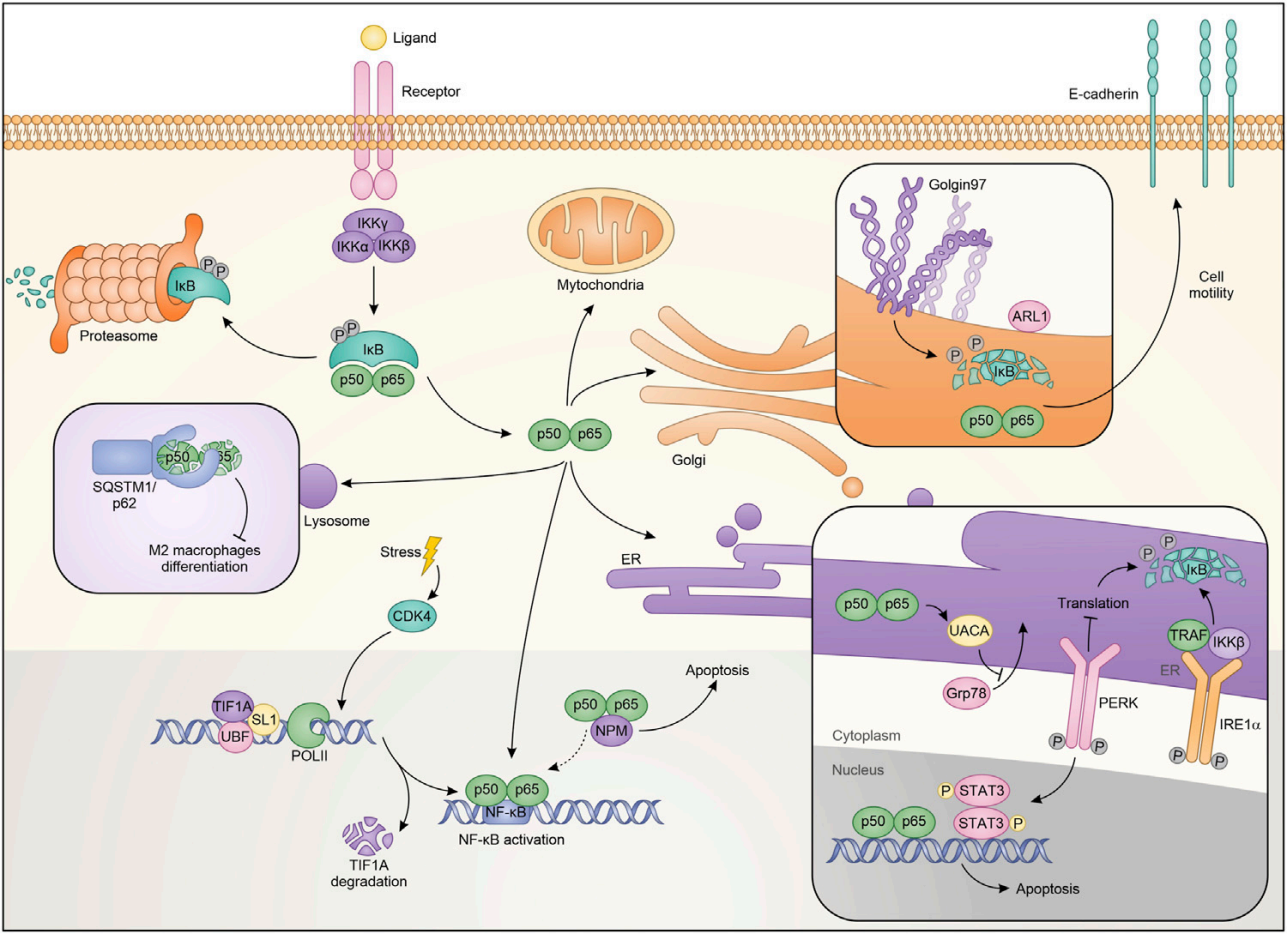
Schematic representation of NF-κB localization and roles in the different cellular compartments.

Cellular stress mechanisms generally cause protein flows from outside to inside organelles and vice versa. During these stressors, the nucleolus is also activated by expelling or trapping a series of regulatory proteins (Audas et al., 2012; Salmina et al., 2017). Nucleolar functions of NF-κB contrast with its pro-oncogene activity, contributing to inhibit growth and inducing cell death under specific stimuli. Specifically, NF-κB was shown to localize into the nucleolus, possibly via the interaction with nucleolar proteins NFBP (Sweet et al., 2003) and nucleophosmin/B23 (NPM) (Dhar et al., 2004), thus causing a reduction in the transcription of anti-apoptotic genes. After aspirin and UV-C treatment, NF-κB relocalizes to the nucleolus in SW480 colon cancer cells through its N-terminal motif and regulates cell apoptosis. Also in this case, sequestering RelA away from its target promoters causes a decrease in the transcription of NF-κB-dependent antiapoptotic genes (Stark and Dunlop, 2005). However, in addition to these actions, RelA mediates apoptosis through NPM dependent modulation of nucleolar pathways (Okuwaki, 2008). Indeed, siRNA against NPM completely abrogates apoptosis mediated by nucleolar RelA (Figure 2) (Khandelwal et al., 2011).

### Role in Stress/Ribosome Biogenesis

NF-κB also plays a significant role in ribosome biogenesis. Pre-ribosomes are generated starting from transcription of ribosomal DNA (rDNA) by a multi-protein complex consisting of RNA polymerase I (PolI), upstream binding factor (UBF), SL-1, TIF-IA and other accessory proteins, generates pre-ribosomes, which are further matured to the 40S and 60S ribosomal subunits (Grummt, 2003). NF-κB plays a critical role in maintaining nucleolar homeostasis under stress (Chen and Stark, 2018). Consistent with this, it was demonstrated that degradation of TIF-IA, in response to stress stimuli like aspirin and UV-C, disrupts PolI complex and activates the cytoplasmic NF-κB functions. Moreover, blocking TIF-IA degradation inhibits stress effects on the NF-κB pathway. Authors identified cyclin-dependent kinase CDK4 as the possible upstream mechanism mediating the degradation of TIF-IA. Consistent with the authors’ hypothesis is the reduced activity of CDK4 following treatment with aspirin or after exposure to UV-C (Thoms et al., 2007a). Therefore, using a CDK4 inhibitor, they demonstrated a substantial decrease in TIF-IA protein levels, identifying a new pathway for TIF-IA inactivation in mediating NF-κB functions in nucleolus. The mechanism by which NF-κB activation occurs is possibly related to multiple pathways, already known in its regulation, and residing within the nucleoli. It is possible that the PolI complex activates CK2 (Morotti et al., 2015) which in turn phosphorylates and promotes the degradation of IκBα (Figure 2) (Chen et al., 2018).

## NF-κB and Endoplasmic Reticulum

The endoplasmic reticulum (ER) is an organelle involved in the regulation of protein synthesis and specifically responsible for their assembly, folding, transport and degradation. The ER stress induces an adaptive program called the unfolded protein response (UPR), which influences the activity of transcription factors and kinase pathways and may play relevant roles in cancer (Wang and Kaufman, 2016). It has been shown that conditions perturbing the ER are responsible for (or require) NF-κB activation (Pahl and Baeuerle, 1997; Kitamura, 2009; Hotamisligil, 2010), but the mechanisms are unknown. In a model of hepato-carcinogenesis driven by accumulation of HBV envelope polypeptides, the lack of canonical NF-κB pathway influences HBV surface-antigen (HBsAg) processing, through the inhibition of UPR. Specifically, HBsAg+/IKK2KDHep mice, characterized by inhibition of canonical NF-κB signaling, showed a decrease of hepatic binding immunoglobulin protein (BiP), one of the main regulators of the folding of new proteins (Hetz, 2012). BiP is crucial for ER protein folding and inhibits the UPR signaling. The decrease in BiP expression results in prolonged ER stress and in CCAAT/enhancer binding homologous protein (CHOP) over-expression, induction of cell cycle arrest, increment of DNA damage, ultimately causing a greater incidence of hepatocellular carcinoma (Sunami et al., 2016). Treatment with ER stress inducers enhances the phosphorylation of IκBα protein in TC-1 and HeLa cells promoting autophagy and apoptosis (Zhu et al., 2017). Additionally, it has been demonstrated that estrogen (E2)-induced apoptosis in E2-deprived breast cancer cells inhibits apoptosis through regulation of NF-κB-ER crosstalk. Ping and collaborators showed that E2 regulates the activation of NF-κB up-regulating C/EBPβ. Mechanistically, E2 induces apoptosis via PERK activation, one of the UPR sensors (Walter and Ron, 2011). Based on these results PERK kinase increases the ability of NF-κB to bind DNA. PERK kinase modulates the nuclear function of NF-κB, through STAT3 activation (Fan et al., 2018). The role of STAT3 in the ER has been extensively described as well as its connection with the NF-κB pathway (Grabner et al., 2015; Avalle et al., 2019). Therefore it is not surprising that the two proteins are connected in ER-mediated apoptosis induction (Fan et al., 2018). Additionally, it was demonstrated that an optimal activation of NF-κB during ER stress requires inputs from both IRE1 and PERK activities. Indeed, it was observed that IRE1 and IKK influence each other with consequent NF-κB modulation. IRE1α −/− cells showed a reduction of the basal IκBα phosphorylation and NF-κB activation. Mechanistically, after the stress factors increase the protein load deployed in the ER there is a massive autophosphorylation which leads to the activation of both IRE1α and PERK. On one hand, IRE1α binds TRAF2 and regulates the protein kinase IKKβ, on the other PERK phosphorylates eukaryotic translation initiation factor eIF2α, causing translation attenuation of multiple proteins, including IκBα (Figure 2). Therefore, both the PERK and IRE1α branches of the UPR work together to modulate the activity of NF-κB. From the therapeutic point of view, activation of this pathway that restores basal IKK/NF-κB activity and concomitantly induces UPRr, could sensitize cells to death stimuli (Tam et al., 2012). In contrast, other studies revealed that NF-κB inhibits UPR up-regulating the expression of UACA which in turn binds PAR-4, preventing translocation of GRP78 from the endoplasmic reticulum to the cell surface. This ultimately results in apoptotic resistance in cancer cells (Burikhanov et al., 2013). In conclusion, there are different levels of basal interaction between the UPR complex and the NF-κB pathway. Balancing these interactions is critical to limit maximal ER stress.

## NF-κB and Lysosome

In addition to the afore mentioned cellular compartments, NF-κB activity also intervenes with lysosomal functions (Liu et al., 2003; Chang et al., 2013). Several evidences have revealed that the lysosome participates in cell death through the release into the cytoplasm of cathepsin B, which in turn activates caspase-dependent or caspase-independent mechanisms of cell death (Ferri and Kroemer, 2001; Foghsgaard et al., 2001). Among the mechanisms by which TNFα induces cell death, it should be mentioned the linking with TNF-R1, which determines the activation of lysosomal enzymes including cathepsin B. Ni Liu and colleagues reported that NF-κB antagonizes this lysosomal mechanism of cell death. These effects were attributed to the NF-κB-dependent upregulation of Spi2A, a specific inhibitor of both serine proteases and lysosomal cysteine cathepsins. Specifically, using RelA^−/−^ MEFs they demonstrate that after TNFα treatment the transcription of Spi2A was abolished. Moreover, the same cells transduced with Spi2A, showed an increase of survival, indicating both that the RelA-mediated protection is due to Spi2A, and that Spi2A overexpression is sufficient per se to inhibit TNFα-induced death (Figure 2) (Liu et al., 2003). Autophagy is one of the main functions mediated by lysosomal complexes. Selective signals are responsible for the initiation of the autophagy, one of which is ubiquitination, that generates ubiquitinylated substrates that are in turns recognized by ubiquitin-binding proteins, such as p62 and SQSTM1 (sequestosome 1) (Mizushima et al., 2008; Chang et al., 2013). In this sense, it has been shown that NF-κB in hepatoma-associated M2-like macrophages forms aggresome-like structures (ALS) which are recognized by the p62/SQSTM1 complex and in which NF-κB is degraded. This event is mediated by TLR2-triggered sustained phosphorylation of ERK1/2 (Chang et al., 2013). The results suggested by the authors indicate that tumors can limit the anticancer activities of TAMs by degrading NF-κB. These findings apparently contrast with the predominant idea that inhibition of the NF-κB in tumor-associated macrophages (TAMs) is a mechanism for inducing antitumor immunity (Mantovani et al., 2002). All the above observations strongly support the idea that the crosstalk between the different factors and signals that positively or negatively regulate the NF-κB pathway in the lysosome can induce, depending on the cellular context, a pro or anti-tumor function.

## NF-κB and Golgi

The Golgi network is the most important sorting node of newly synthesized proteins and, therefore, it is involved in various cellular processes, including the genesis of organelles, the modulation of cytoskeleton, the activation of receptor signals as well as cell apoptosis and cell cycle progression (Derby and Gleeson, 2007). The crosstalk between NF-κB and Golgi relies heavily on the inhibition of golgin-97, a protein associated with the Golgi apparatus, and necessary for the maintenance of its architecture (Barr and Short, 2003). Indeed, golgin-97 down-regulation induces NF-κB activation by reducing IκBα levels. Particularly, Hsu and colleagues proposed that, under physiological conditions, golgin-97 can interact with some still not identified proteins and regulate IκBα level (Figure 2). In breast cancer, the downregulation of IκBα and the consequent activation of NF-κB, induces EMT and the secretion of molecules that promote cell migration and motility (Chua et al., 2007; Pires et al., 2017; Hsu et al., 2018). However, the study did not exclude the possibility that NF-κB activation depends on ER stress induced by protein missorting or unfolding due to golgin-97 down-regulation. The identification of such unusual NF-κB activity sheds light on alternative mechanisms to promote cancer progression and metastasis formation. In this view, golgin-97 can represent a new biomarker able to direct therapy toward treatments with NF-κB inhibitors.

## NF-κB as a Therapeutic Target

Given its role in cancer and other pathologies, the notion that NF-κB should be a therapeutic target is very popular among the experts in the field. Indeed, several drugs that suppress NF-κB as well as inhibitors of its downstream targets or upstream activators have been proposed (Orlowski and Baldwin, 2002; Li et al., 2015). Since the nature of the NF-κB target genes, often associated with pro-tumor pathways, the main therapeutic approaches are essentially aimed to its inhibition (Davis et al., 1999; Adams, 2001; Pikarsky and Ben-Neriah, 2006; Arora et al., 2014; Nomura et al., 2016). Several compounds have been proposed to block IκBα degradation and therefore to induce NF-κB nuclear localization, including proteasome inhibitors, peptides and drugs that reduce IκB kinase (IKK) activity (Keifer et al., 2001; Yamamoto and Gaynor, 2001; Hideshima et al., 2002). However, NF-κB activation has been proved to be essential to induce cell death in some cellular settings. Indeed, several studies suggest that NF-κB is required for example for Taxol and hydrogen peroxide-induced cell death (Huang et al., 2000; Ryan et al., 2000). An important aspect to be considered for therapies based on the inhibition/activation of NF-κB, is the interdependence of this pathway with the different cellular organelles. Specific pharmacological combinations are capable of inhibiting the NF-κB pathway in different cellular compartments. For example, IKK and PERK inhibition targets NF-κB activity makes renal carcinoma cells more prone to apoptosis. Different inhibitors of PERK pathway may be used to blunt NF-κB activation. PERK inhibitor, GSK2656157, decreases the activity of the p65 subunit by inhibiting ATF4 induction (Iwasaki et al., 2014). Salubrinal, a selective inhibitor of eIF2α dephosphorylation, inhibits TNFα-induced activation of NF-κB (Nakajima et al., 2015). Ghosh et al. described an inhibitor that prevents the oligomerization of IRE1α, thereby suppressing its activities. It will be helpful to evaluate whether this inhibitor has consequences on the NF-κB pathway (Ghosh et al., 2014). These pharmacological approaches provide a link between ER stress and NF-κB although their mechanisms and specificity need to be further evaluated. Concerning the targeting NF-κB with respect to its activity in the different cellular compartments, we demonstrated that targeting or silencing IκBα with PSA in the IκBα-overespressing lung cancer cells, significantly induce mitochondria OXPHOS and increased ROS content and release, correlating with a significant increased sensitivity to apoptotic cell death (Carra et al., 2021). Moreover, it has been demonstrated that IκBα knock-down sensitizes cells to apoptosis and reduces tumor burden in a cancer mouse model. This depends on the ability of IκBα to stabilize the complex between HKII and VDAC1 in the outer mitochondrial membrane, blocking Bax association to VDAC1 and the release of cytochrome C (Pazarentzos et al., 2014). In different contexts NF-κB shows antiapoptotic activity and therefore its inhibitor IκBα exerts a pro-apoptotic function. However, many studies demonstrate that more aggressive tumor cells show concomitant NF-κB activity and increased expression of inhibitor IκBα (Shukla et al., 2004; Sethi et al., 2008). It is thus possible to hypothesize that in this context the anti-apoptotic function of IκBα may prevail and that IκBα targeting/silencing may represent a successful approach to promote cancer cell apoptosis. Considering NF-κB as an oncogene, the loss of its normal nuclear compartmentalization and function could be exploited to inhibit the transcription of anti-apoptotic factors. In this respect, aspirin and UV-C in SW480 colon cancer cells induce accumulation of NF-κB in the nucleoli and reduction in its anti-apoptotic target genes expression (Stark and Dunlop, 2005). Given that PolI silencing induces NF-κB (RelA) displacement to the nucleolus (Stark and Dunlop, 2005), the PolI inhibitor BMH-21 may be used to sensitize cancer cells to apoptosis (Peltonen et al., 2014). On the same line, CDK4 inhibitor, already in clinical use as anti-cancer treatment, induces TIF-IA degradation, NF-κB recruitment to the nucleolus and impairs the NF-κB -dependent expression of anti-apoptotic genes (Thoms et al., 2007b). Lastly, in a multi-targeting approach, the inhibition of NF-κB by activators of the lysosomal degradation pathways could act not only on the tumor component itself, but indirectly on the role of NF-κB in the regulation of the tumor microenvironment (Chang et al., 2013). Although several inhibitors or activating compounds have been proposed in cancer therapy, their efficacy, specificity, and side effects still represent significant issues to be addressed. Overall, the recent findings of NF-κB pathway underline the necessity of combination therapies, that consider its complex interdependence with different cellular compartments (Chang et al., 2013).

## Conclusion

The genes transcribed by RelA/NF-κB are critical and limiting factors in the control of cell survival but also of the apoptotic response mediated by various stimuli (Baldwin, 2001). NF-κB different functions, often even contradictory, can surely be explained by its ability to activate different target genes in different types of cells and conditions. This peculiar and sometimes paradoxical role of NF-κB is now appreciated between its supporters, but it took a long time for it to be revealed. Only in recent years, it became clear that the ability of NF-κB to switch from oncogenic to tumor suppressor functions, have been underestimated. The data discussed above clearly show that NF-κB acts in an unconventional way, outside the nucleus, regulating the most disparate functions in different cellular compartments, often independently of its transcriptional activity and based of the cellular context where it is expressed. In this review, we looked at studies on the function of NF-κB in regulating the activity of different cellular compartments, and it appears that research in this area is on the rise. For example, this is particularly evident in the study of the mitochondrion, being an essential organelle that drives cellular homeostasis. NF-κB, localized in mitochondria or via crosstalk with mitochondrial components, regulates a myriad of cellular functions such as apoptosis, cell survival and mitochondrial gene expression. NF-κB activity in other cellular compartments, such as the Golgi apparatus, is less investigated and deserves further attention. However, a crucial point is now to understand how “non-canonical” functions of NF-κB can be tuned to maximize therapeutic treatments and, specifically, activate or inhibit NF-κB, depending on the role it plays (onco gene/suppressor) in each specific context. Furthermore, it is necessary to consider the precarious balance and the easy versatility of NF-κB in switching from its function of tumor suppressor to the oncogenic functions. The role of NF-κB signaling in maintaining cellular homeostasis in several cell types must be also considered. Indeed, loss of NF-κB p50/p105 has been shown to impair innate and adaptive immune functions (Beinke and Ley, 2004). The absence of p52/p100 or c-Rel, lead to a lack of development of lymphoid structures, with impaired maturation and function of B and T lymphocytes (Köntgen et al., 1995; Franzoso et al., 1998). Moreover, RelA knockout mice are lethal due to extensive apoptosis in embryonic hepatocytes (Beg et al., 1995). Therefore, a better definition of the NF-κB pathway and its crosstalk will be fundamental to identify more specific therapeutic approaches. As a further layer of complexity on the role of NF-κB in various organelles, it should be evaluated whether NF-κB is involved at the basal level or only upon stimulation. While most of the reports have shown that various stimulation argument the function of NF-κB in various cellular compartments, it should be noted that even basal NF-κB seems to play a critical role besides the transcriptional level. In this review, we have highlighted the concept of a pool of NF-κB molecules, that can be basally located in various organelles, where it may play a stably role, like through the interaction with NPM in the nucleoli, or in the mitochondria through the binding of ANT. In line with these considerations, we may also speculate that some of the phenotypes that are observed in KO mice could belong to such unknown functions inside various organelles. Further investigations in the field are indeed mandatory, since they may reveal potentially relevant therapeutical implications.
